# Fine Mapping of the Pond Snail Left-Right Asymmetry (Chirality) Locus Using RAD-Seq and Fibre-FISH

**DOI:** 10.1371/journal.pone.0071067

**Published:** 2013-08-12

**Authors:** Mengning Maureen Liu, John W. Davey, Ruby Banerjee, Jie Han, Fengtang Yang, Aziz Aboobaker, Mark L. Blaxter, Angus Davison

**Affiliations:** 1 School of Biology, University of Nottingham, University Park, Nottingham, United Kingdom; 2 Department of Plant Sciences, University of Cambridge, Cambridge, United Kingdom; 3 Institute of Evolutionary Biology, University of Edinburgh, Edinburgh, United Kingdom; 4 Department of Zoology, University of Cambridge, Cambridge, United Kingdom; 5 Wellcome Trust Sanger Institute, Wellcome Trust Genome Campus, Hinxton, Cambridge, United Kingdom; 6 College of Life Sciences, Beijing Normal University, Beijing, P. R. China; 7 Department of Zoology, University of Oxford, Oxford, United Kingdom; 8 The GenePool Genomics Facility, School of Biological Sciences, University of Edinburgh, Edinburgh, United Kingdom; Auburn University, United States of America

## Abstract

The left-right asymmetry of snails, including the direction of shell coiling, is determined by the delayed effect of a maternal gene on the chiral twist that takes place during early embryonic cell divisions. Yet, despite being a well-established classical problem, the identity of the gene and the means by which left-right asymmetry is established in snails remain unknown. We here demonstrate the power of new genomic approaches for identification of the chirality gene, “*D”*. First, heterozygous (*Dd*) pond snails *Lymnaea stagnalis* were self-fertilised or backcrossed, and the genotype of more than six thousand offspring inferred, either dextral (*DD*/*Dd*) or sinistral (*dd*). Then, twenty of the offspring were used for Restriction-site-Associated DNA Sequencing (RAD-Seq) to identify anonymous molecular markers that are linked to the chirality locus. A local genetic map was constructed by genotyping three flanking markers in over three thousand snails. The three markers lie either side of the chirality locus, with one very tightly linked (<0.1 cM). Finally, bacterial artificial chromosomes (BACs) were isolated that contained the three loci. Fluorescent *in situ* hybridization (FISH) of pachytene cells showed that the three BACs tightly cluster on the same bivalent chromosome. Fibre-FISH identified a region of greater that ∼0.4 Mb between two BAC clone markers that must contain *D*. This work therefore establishes the resources for molecular identification of the chirality gene and the variation that underpins sinistral and dextral coiling. More generally, the results also show that combining genomic technologies, such as RAD-Seq and high resolution FISH, is a robust approach for mapping key loci in non-model systems.

## Introduction

Consistent left-right asymmetry is a decisive feature of embryonic development, yet the breaking of bilateral symmetry is still poorly understood [1]. Until recently, the most popular model for this process was that motile cilia create a chiral extracellular fluid flow during gastrulation. However, a body of research points towards a symmetry-breaking event that occurs much earlier at the intracellular level [1]. In the hypothetical view of Brown and Wolpert [2], the general solution to the problem of symmetry breaking is provided by a pre-existing asymmetric molecular reference, or “F-molecule”, which aligns with anterior-posterior and dorsal-ventral axes and creates an asymmetric signal, perhaps by then transporting an effector molecule towards the left or right. Asymmetry is thus entirely dependent upon the chirality and alignment of the F-molecule.

Pond snails of the genus *Lymnaea* have been used to study asymmetry for nearly 120 years. It has been known since 1894 that both the coil of the shell and the entire body asymmetry of a snail are entirely predicated by the spiral twist that takes place as the 4-cell embryo divides [3]. Later, Boycott and Diver [4] observed that shell coiling in the pond snail *L. peregra* is a hereditary character, but reported that the patterns of variation in the offspring were difficult to understand, requiring a complicated model to fit the data. Sturtevant [5] immediately hypothesised that these odd patterns were because the expression of the gene is delayed by a generation, an “inspired guess” [6] that proved to be correct. In most snails that have been examined, the chromosomal locus that determines asymmetry acts via a maternal effect [7]. In dominant dextral-coiling *L. stagnalis* (genotype *DD* or *Dd*) the movement of cells in the early embryo, and the twist of the adult animal’s shell is clockwise, whereas in genetically sinistral (recessive, *dd*) snails it is anticlockwise.

However, even though the early research on *Lymnaea* has an important role in the narrative of Mendelian genetics [8], is a staple item in text books [9], and continues to influence current thinking [1], pond snails have until recently been relatively neglected. Little progress on understanding asymmetry in *Lymnaea* was made in the latter twentieth century. The most revealing result was that the maternal inheritance of the gene is apparently determined by a factor that the mother deposits in the unfertilized egg [10]. It is presumed that it is this factor, or an intermediate, that then goes on to direct the dynamics of later chiral blastomere division [11]. It has also been shown that the early development in sinistrals is not an exact mirror image of development in dextrals [12], with many sinistral embryos failing to develop at all [13], [14]. In the last few years, the work on *Lymnaea* has been given added impetus because it has been discovered that a common gene, *nodal*, links deuterostome and molluscan asymmetry, although in neither case is it the earliest symmetry breaking determinant [11], [15].

The primary motivation for using *L. stagnalis* is that asymmetry is established very early, and the problem is genetically tractable, because both chiral forms exist. However, there are few genomic resources currently available for use in this species [16], [17]. We have therefore utilised a new and transformative high-throughput DNA sequencing technology, Restriction-site-Associated DNA Sequencing (RAD-Seq) [18], [19], to find anonymous markers that flank the chirality locus in *L. stagnalis* directly. We identified 20 markers linked to the locus. Further investigations in a large crossing panel using a subset of these markers showed that the markers flank the chirality-determining *D* locus and that one is very closely linked, both by recombination and in physical distance. We pinpointed the location of the chirality gene with high resolution pachytene fluorescent *in situ* hybridisation (FISH) and fibre FISH, demonstrating that these are robust techniques for high resolution physical mapping in snails. Together, these results set in place the resources for identification of the chirality gene itself.

To avoid confusing terminology, snail shell coiling or chirality phenotypes are “dextral” or “sinistral”, whereas equivalent chromosomal genotypes are either *DD*, *Dd* (“genetically dextral”; dextrality is dominant) or *dd* (“genetically sinistral”). Similarly, for alleles of anonymous loci that are in linkage with the chirality genotype, alleles originating from the dextrally-derived chromosome are represented in upper case and alleles from the sinistrally-derived chromosome are lower case.

## Results and Discussion

### Generation of a Large Mapping Cross

**Figure 1:**
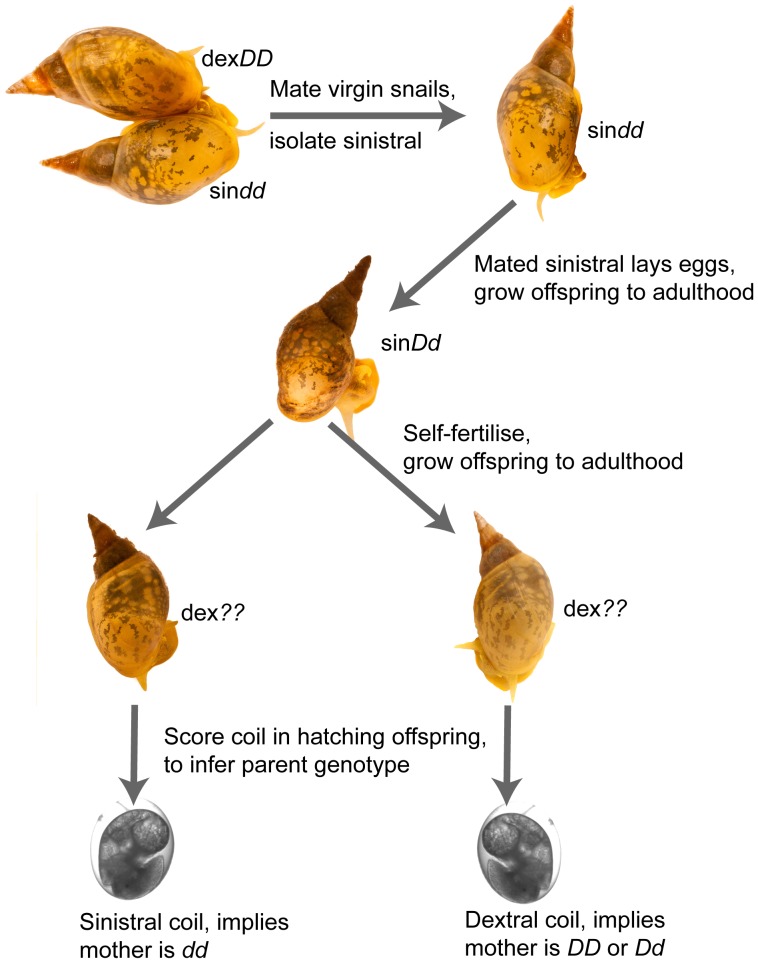
Schematic of crossing strategy used to generate snails that segregate for chirality genotype. Genotypes of snails are labelled as *DD*, *Dd* and *dd*, with the phenotypes labelled as sin and dex. The phenotype of offspring is determined by the maternal genotype (see main text).

*L. stagnalis* are simultaneous hermaphrodites but outcross in preference. To generate snails for the mapping, we obliged virgin *Dd* snails to self-fertilise (Figure 1). The genotype of 5949 offspring was inferred, by the direction of coiling of their offspring, of which 4513 were genetically dextral (*DD* or *Dd*) and 1436 sinistral (*dd*). This ratio does not deviate significantly from the 3∶1 expected (*Χ* ^2^ = 2.355; *P*<0.125). A lesser number of snails were generated by backcrossing *Dd* heterozygotes to *dd* snails, with the *Dd* snail “father” being removed after mating and the *dd* used as the “mother”. The genotype of 389 offspring was inferred, of which 221 were genetically dextral (*DD* or *Dd*) and 168 sinistral (*dd*). This ratio differed from the 1∶1 expected (*Χ* ^2^ = 7.221; *P*<0.007). As it has previously been shown [13], [14] that a high proportion of embryos fail to develop from a mother that is genetically sinistral, *dd*, then we speculate that one explanation is differential mortality of *dd* individuals compared with *Dd* individuals from a common *dd* mother.

### Discovery of Twenty RAD Tags in Linkage with the Snail Chirality Locus

We prepared a RAD-Seq library from genomic DNA of twenty individuals, all offspring of the same self-fertilised snail, genotype *Dd,* using the 8-base cutter SbfI. Ten of the snails were of genotype *DD* or *Dd* (genetically dextral), with the other ten of genotype *dd* (genetically sinistral). Assuming a haploid genome size of 1.19 Gb [20] and a 37% GC content [21], SbfI was predicted to have in the region of ∼9467 cut sites, and thus generate ∼18934 RAD tags. After generating 18.5 million Illumina GAIIx 101 bp read pairs from this library, RADtools [22] was used to identify 62758 candidate alleles at 52124 candidate loci, with a mean depth of 24 reads per locus (SD 6; Table S1). The excess number of RAD tags over the expected could be for a variety of reasons e.g. sequencing error and/or more SbfI sites than expected [23].

To identify putative chirality-linked loci, we searched for candidate loci with an allele present in all genetically dextral (*DD* or *Dd*) snails and absent in all sinistral individuals (putative *D* alleles), with the complementary marker (putative *d* alleles) present in all sinistral individuals and some dextral (presumably *Dd*) snails. This analysis recovered twenty candidate loci, with all twenty putative sinistral (*d*) alleles present in the same seven dextral snails (presumed genotype *Dd*) and absent in the remaining three (presumed genotype *DD*). The twenty markers were in complete linkage with the chirality locus in these twenty test individuals.

Paired-end RAD reads [24] from these twenty markers were assembled into long contigs (mean length 473 bp) to identify polymorphisms between dextral and sinistral haplotypes that would be suitable for development as PCR markers (Table S2). We initially focussed our efforts on five markers, rad1, rad2, rad4, rad5 and rad7, because dextral and sinistral chromosome-derived alleles for these markers differed by an insertion-deletion polymorphism, which made developing PCR assays to score large numbers of snails simpler. Using these PCR assays, we found that the inferred genotypes of the original twenty snails were perfectly validated for all loci tested, confirming that the bioinformatically inferred genotypes were correct. Using a notation where dextral and sinistral chromosome-derived alleles are upper and lower case respectively, we confirmed that the majority of genetically sinistral snails are of genotype *rad1/rad1 rad2/rad2 rad4/rad4 rad5/rad5 rad7/rad7*. The majority of dextral snails are of genotype *RAD1/RAD1 RAD2/RAD2 RAD4/RAD4 RAD5/RAD5 RAD7/RAD7* or *RAD1/rad1 RAD2/rad2 RAD4/rad4 RAD5/rad5 RAD7/rad7*, presumably corresponding to genetically *DD* or *Dd* snails, respectively.

### One RAD Tag is Less than 0.1 Centimorgans (cM) from the Chirality Locus

**Figure 2:**
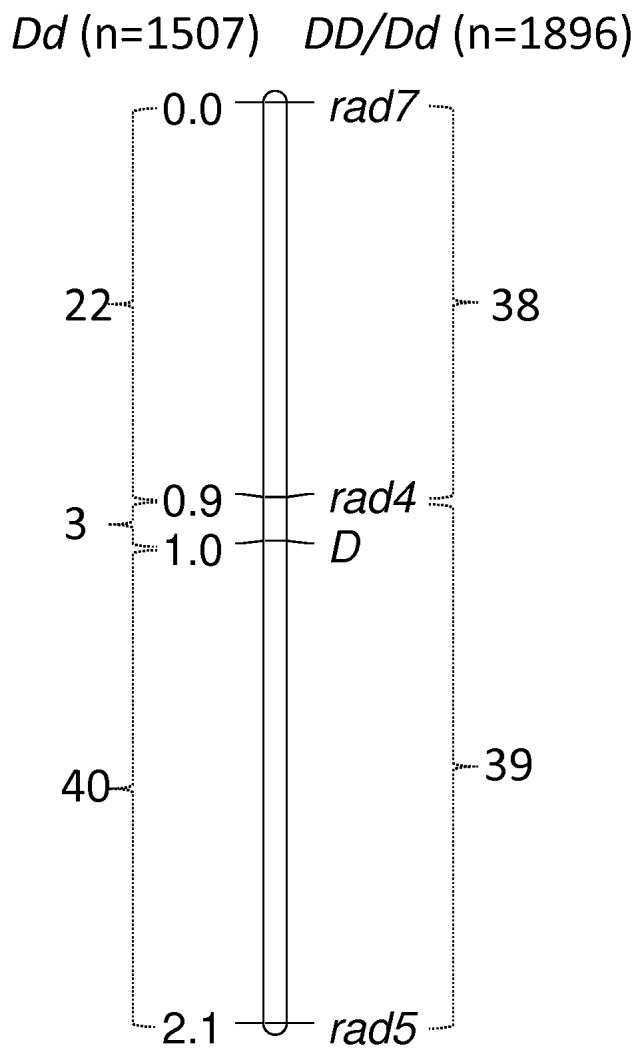
Genetic map of the region containing the chirality locus, *D,* with numbers of recombinants recovered from genetically sinistral (*dd*) and dextral snails (*DD* or *Dd*.) It was not possible to determine the precise number of recombinants in the interval between marker rad4 and chirality locus D in *DD* or *Dd* snails, because of uncertainty as to the chirality genotype of recombinants.

Three markers were scored in 3403 snails, both genetically sinistral (*dd*) and dextral (*DD or Dd*), enabling the construction a local genetic map for the chirality locus (Figure 2). In all, 1507 genetically sinistral snails were scored for RAD-seq markers 4, 5 and 7, yielding 65 putative recombinant-chromosome containing individuals (Table 1). One of these three loci, rad4, was within 0.1 cM of the chirality locus *D*, with only three recombinants in 1507 snails discovered. The other two loci were also closely linked to the chirality locus, rad7 being on the same side as rad4 (25 recombinants) and rad5 being on the other side (40 recombinants). Similar numbers were found in dextral snails: 1896 genetically dextral snails were scored, yielding 77 recombinant individuals for the three loci, 38 between rad4 and rad7, and 39 between rad4 and rad5. In the dextral snails, it was not possible to determine the number of recombinants between the chirality locus *D* and other loci, because of uncertainty regarding their precise genotype (*DD* or *Dd*).

**Table 1:** Summary of genotypes of all individuals.

Number of snails	rad7	rad4	Chirality genotype	rad5	Recombinant between?
**Dextrals**					
1233	*RAD7/rad7*	*RAD4/rad4*	*Dd*	*RAD5/rad5*	Not recombinant
586	*RAD7/RAD7*	*RAD4/RAD4*	*DD*	*RAD5/RAD5*	Not recombinant
15	*RAD7/RAD7*	*RAD4/rad4*	*DD*/*Dd*	*RAD5/rad5*	*rad4* and *rad7*
10	*RAD7/rad7*	*RAD4/RAD4*	*DD*/*Dd*	*RAD5/RAD5*	*rad4* and *rad7*
13	*rad7/rad7*	*RAD4/rad4*	*DD*/*Dd*	*RAD5/rad5*	*rad4* and *rad7*
0	*rad7/rad7*	*rad4/rad4*	*Dd*	*RAD5/rad5*	*rad4* and *D*
14	*RAD7/rad7*	*RAD4/rad4*	*Dd*	*rad5/rad5*	*D* and *rad5*
12	*RAD7/rad7*	*RAD4/rad4*	*DD*/*Dd*	*RAD5/RAD5*	*rad4* and *rad5*
13	*RAD7/RAD7*	*RAD4/RAD4*	*DD*/*Dd*	*RAD5/rad5*	*rad4* and *rad5*
**Sinistrals**					
1442	*rad7/rad7*	*rad4/rad4*	*dd*	*rad5/rad5*	Not recombinant
22	*RAD7/rad7*	*rad4/rad4*	*dd*	*rad5/rad5*	*rad4* and *rad7*
3	*RAD7/rad7*	*RAD4/rad4*	*dd*	*rad5/rad5*	*rad4* and *D*
40	*rad7/rad7*	*rad4/rad4*	*dd*	*RAD5/rad5*	*D* and *rad5*
**Total = 3403**					

Two additional markers were scored in fewer individuals, and then not used further. Locus rad1 was discarded because no recombinants were discovered between it and rad7 (*n* = 472). Locus rad2 was also not used further because no recombinants were discovered between it and rad5 (*n* = 42). SNP and restriction site polymorphism genotyping assays (not shown) were subsequently developed for the other markers. No evidence that any of them were more closely linked to the chirality locus than rad4 or rad5 was found, and they were not used further.

The combined genotypes allowed us to infer the precise chirality genotype of the majority of the genetically dextral snails (excepting those snails that show evidence of recombination in that region). There was no evidence of segregation distortion in genetic dextrals: 1233 *DD* snails were inferred versus 586 *Dd* snails, not significantly different from the expected 2∶1 ratio (*Χ* ^2^ = 1.022; P<0.312).

To give some possible clue as to the identity of the genes that may be contained within this region, we carried out BLASTX analyses of the anonymous RAD-Seq tags. The rad7 tag had a strong hit (expect value (E) of 1e-21) to the probable E3 ubiquitin-protein ligase, HERC2. We do not consider this putative orthologue a strong candidate gene, because the rad7 locus is 1 cM away from the chirality locus, though long distance regulation of gene expression is possible, including an example involving HERC in humans [25], [26]. The other loci did not have any good BLAST hits.

### The Physical Distance between RAD Tag Markers that Flank the Chirality Locus is Greater than Around 0.4 to 0.6 Mb

With a view to identifying the chirality locus in the future, we wished to estimate the physical distance between the two loci rad4 and rad5, and explore synteny with other mollusc genomes. Existing genomic resources are not just poor for *L. stagnalis*, but for molluscs in general. While there are two published molluscan genomes (*Lottia gigantea*, and *Crassostrea gigas* [27], [28]), and two others are publicly available (*Biomphalaria glabrata* and *Aplysia californica* (http://biology.unm.edu/biomphalaria-genome/; http://smithlabdb.usc.edu/cgi-bin/hgGateway) their long-range assembly has proved problematic. The recent comparative analysis of three spiralian genomes showed that regions of macro and microsynteny do exist [28], but the HERC gene does not seem to be in a region of conserved synteny. Analysis of the other RAD-Seq tags did not show any pattern consistent with synteny between *L. stagnalis* and the closest sequenced relative, the heterobranch gastropod *B. glabrata*. This does not mean that synteny does not exist in the chirality region, just that we have not yet discovered evidence for it.

**Figure 3:**
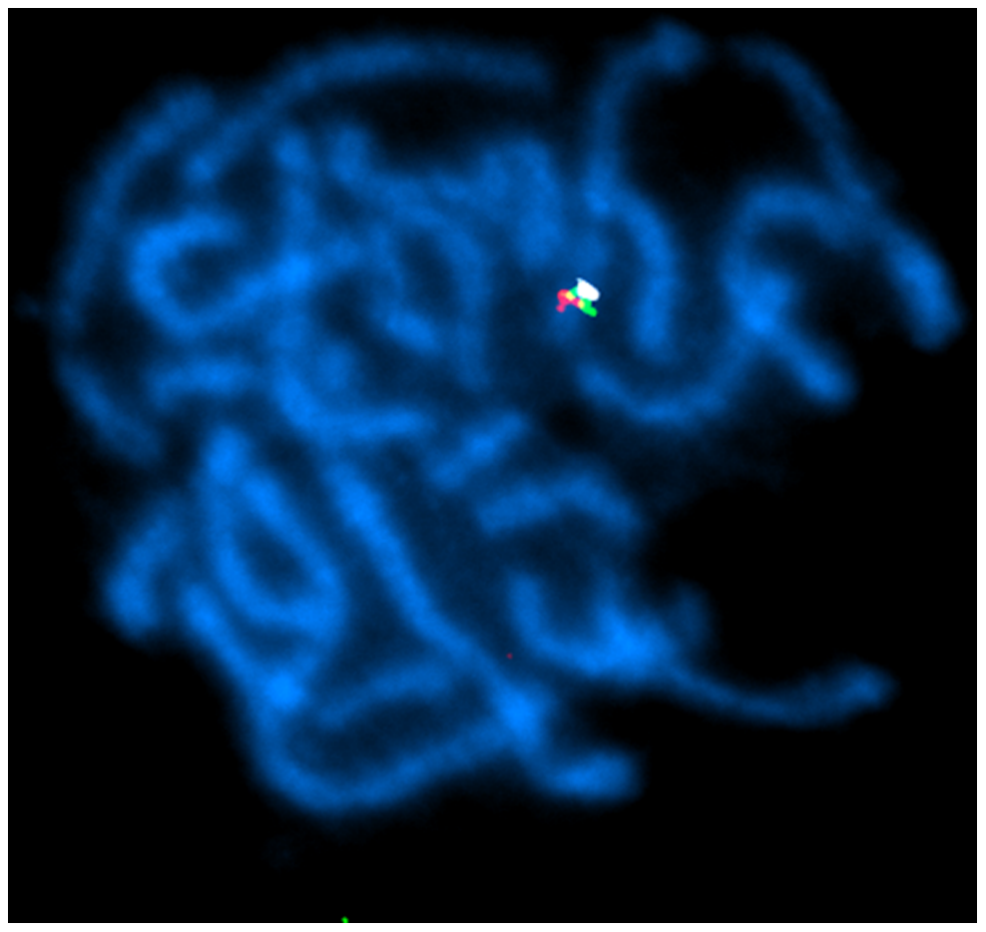
Pachytene-FISH mapping of the chirality locus. Pachytene-FISH was performed using BACs containing RAD-Seq locus rad7 (white; BAC clone G1A, 137 kb), rad4 (red; R6F, 129 kb) and rad5 (green; B2D, 97 kb). All three BACs show hybridisation to the same bivalent chromosome. Chromosomes are counterstained with 4′,6-diamidino-2-phenylindole (DAPI; blue).

**Figure 4:**
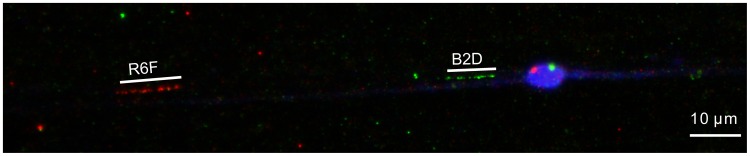
Fibre FISH using BACs that bound the chirality locus. The gap between the hybridisation signal from BAC clones containing loci rad4 (red, R6F, 129 kb) and rad5 (green, B2D, 97 kb) is at least 4 to 5 times the length of a BAC clone. The preparation is counterstained with 4′,6-diamidino-2-phenylindole (DAPI; blue). In this example from a genetically dextral snail, an interphase, meiotic cell is also visible, showing signal from hybridisation to both BACs.

In theory, cytogenetic approaches can anchor accumulating genomic data, but in molluscs the most useful technique, fluorescent *in situ* hybridisation (FISH), has been limited to use on cell lines of *B. glabrata* [29], [30]. We therefore developed FISH methods to visualise individual loci on the chromosomes of *L. stagnalis*, using non-cultured live cells. First, separate clones containing the loci rad4, rad5 and rad7 were retrieved from a bacterial artificial chromosome (BAC) library. Pachytene FISH was used to confirm that BACs containing rad4, rad5 and rad7 co-localise to the same bivalent chromosome (Figure 3). Fibre-FISH was then used to try to determine the physical distance between the two markers that bound the chirality locus (rad4, rad5). Unfortunately, few intact fibre-FISH images were obtained (n = 4). This is most likely because the majority of fibres had been broken, because of the relatively great distance between the BACs, meaning that the syntenic relationship between the two BACs could only be visualised in relatively unstretched chromatin fibres and interphase nuclei. The latter were rare in our preparation. These reasons, together with inconsistencies in the stretching level of extended chromatin fibres, mean that it is not possible to define a precise upper bound for the chirality region. Nonetheless, using the estimated size of the BACs (from gel electrophoresis) as a scale, we assessed that the distance between the extreme ends of the BACs is approximately 0.4 to 0.6 Mb (Figure 4). This puts a lower bound of the size of the gap between the markers. As we detected 82 crossovers in this region (Table 1), we will in the future be able to precisely define the non-recombined haplotype, and thus the segment of the genome that contains the *dd* allele, and thus the chirality locus.

We expected to be able to sample the genome at each SbfI restriction site, or about every 250 kb of the genome, yielding ∼9.4×10^3^ tags marking ∼18.9×10^3^ anonymous loci. While we recovered many RAD tag sites from the experiment, only a few of these were in linkage with the chirality locus *D*, and mapping of BAC clones containing the most closely mapped markers yielded an interval of at least ∼0.4–0.6 Mb. We speculate that other SbfI sites within this region might not be detectable independently because they are invariable, or lie in repeat regions in the repeat-rich genome [23]. The FISH data both affirm the correctness of the RAD-Seq genetic mapping, and also provide a framework for the isolation of the complete sequence of the recombination interval through additional mapping and BAC clone walking.

### Conclusions

We have shown how RAD-Seq is a transforming technology in that a single library derived from 20 snails has enabled us to go from resource poor non-model organism to developing thousands of polymorphic markers. The markers were then used to isolate tags linked to an important maternal effect gene, from which custom markers were used to create a genetic recombination map and also correlate recombination to physical distance. High-resolution pachytene-FISH together with fibre-FISH are robust techniques for high resolution physical mapping. After a century of speculation, we can now rapidly home in on the long-sought snail chirality gene.

## Materials and Methods

### Ethics Statement

All animal work was conducted according to relevant national and international guidelines. No specific permissions are required to work with invertebrates in the UK. Similarly, no specific permissions were required for the collection of snails from sample sites because they were not collected from protected areas of land. The pond snail, *L. stagnalis*, is not an endangered or protected species.

### Organisms and Crosses

Sinistral snails were a generous gift from Joris Koene and derive from the same source as others have used [11], [31]. A genetically distinct population of dextral snails was sourced from a pond within the University Park, University of Nottingham. Both populations were maintained as previously described [32]. To create the snails for RAD-Seq (see Figure 1), a virgin sinistral *Dd* individual was self-fertilised, then 6338 dextral offspring raised to adulthood. The chirality genotype of these snails was then determined, by scoring the phenotype of their offspring. The expectation was for snails of genotype *DD* or *Dd* (yielding dextral babies) or *dd* (sinistral babies) in a ratio of 3∶1. The advantage of using a self-fertilisation crossing strategy is that only two alleles per locus may be present in the entire segregating generation, potentially of benefit to the bioinformatic analysis of the RAD-Seq data. In addition, in scoring *dd* offspring snails from a self-fertilisation, both chromosomes are potentially recombinant, so there is greater efficiency in scoring molecular markers in the downstream analyses.

### RADSeq Library Construction, Sequencing and Bioinformatics

The paired-end RAD-Seq library was constructed using the SbfI restriction enzyme, following standard procedure [33], including ten dextral individuals (genotype *DD* or *Dd*) and ten sinistral individuals (genotype *dd*). The library was sequenced at the GenePool Genomics Facility, University of Edinburgh (http://genepool.bio.ed.ac.uk), with sequences available at the European Nucleotide Archive, with accession ERP002339.

Briefly, raw reads were separated by individual using the RADpools tool in RADtools v1.2.1 [22], candidate loci were inferred for each individual using RADtags with a cluster distance of 8, and loci were merged across all 20 individuals with RADmarkers, only merging clusters containing identical alleles across individuals and allowing no mismatches. Alleles absent in all sinistral snails and present in all dextral snails were identified, allowing one dextral snail to be absent due to the low read counts for some dextral snails. Twenty four candidate alleles fitted this pattern, 20 of which had a matching allele present in all sinistral snails and seven dextral snails. RADtools clustered 18 of these allele pairs as candidate loci, but two allele pairs were left unclustered by RADtools and were manually paired by sequence similarity. No matching allele could be found for the remaining four candidate alleles and so these were discarded. It may be that there is variation in the Sbf1 recognition site at these loci, and so an alternative allele is absent. Paired end reads for each allele were assembled using VelvetOptimiser v2.1.0 [34] in parallel on the Edinburgh Compute and Data Facility compute cluster (ECDF, http://www.ecdf.ed.ac.uk/, which is partially supported by the eDIKT Initiative http://www.edikt.org.uk).

### Validation of RAD-Seq Markers and Linkage Map

Standard PCR was carried out using Amplitaq Gold polymerase (Invitrogen), 1.5 mM MgCl_2_, and the following cycling conditions: 95°C for 10 min, followed by 35 cycles of 95°C for 30 s, 58°C for 30 s, and 72°C for 1 min. Primers were designed for each locus using Primer 3v.0.4 [35], specifically : **rad1** 5′-TGCTGAAACAGGAATGGACA-3′ and 5′-TGTCTCTGCCACAGAACAGG-3′; **rad2** 5′-CACAAAACAGAAAATGTTCTACTTGAC-3′ and 5′-TTTCTTCTTATCAGAATTATTGCATGT-3′; **rad4** 5′-GAGGAGAGGTTTGATTTCATTGAT-3′ and CATTCCGCAAACTCTCCATT-3′; **rad7** 5′-TCGTCACAGGTTGGTAAACAAG-3′ and ACCTGGTCAACAGCATCTTTGT-3′; **rad5** 5′-TCACAACAGCGTATGGTTGG-3′ and 5′-CGAACATTAGAACTGAGGAACTCG-3′. Dextral and sinistral chromosome-derived alleles for these five PCR products differ by an insertion-deletion polymorphism. Genotypes were therefore inferred by scoring variation in the length of PCR products, following electrophoresis in 2–3% agarose gels. Genotype scores were imported into JoinMap v4.1 (Kyazma) and the linkage map viewed using MapChart 2.2 [36].

### BAC Library

A 10-fold coverage BAC library was constructed commercially in the vector pIndigoBAC-5 by Bio S&T Inc. (Montreal, Canada), using as starting material a mixture of *DD*, *Dd* and *dd* snails. BACs containing RAD tag loci were identified by bulk PCR. DNA prepared from isolated BACs was digested with NotI and the inserts sized using pulse field gel electrophoresis.

### Fluorescent *in situ* Hybridisation

Adult snail ovotestis, liver tissue and albumen gland was used as a source of chromosomes. Cells from tissues were released by repeated pipetting until a cell suspension was obtained. The cell suspension was then centrifuged at 1200 rpm for 5 min in a Heraeus Labofuge 400R, supernatant was removed and the pellet resuspended in 10 ml of 0.56% KCl for 12 min. A few drops of freshly made Carnoy’s fixative (three volumes methanol, one volume glacial acetic acid) were added to the suspension before spinning down cells again at 1200 rpm for 5 min. The supernatant was discarded and the pellet resuspended in freshly made fixative. Cells were spun down at 1200 rpm for 5 min. After two further washes in fixative, cells were resuspended in an appropriate amount of fixative. Meiotic and mitotic cells were together dropped onto sonicated, ethanol washed, air dried slides.

Preparation of target DNA and three BAC probes for meiotic (pachytene) chromosomes, followed by hybridisation, was carried out following the standard FISH protocol with modifications [34], [35] using tissue sourced from both dextral and sinistral snails. Pachytene slides were prepared the night before setting up the hybridisation and aged in a sealed box with a dessicant at room temperature. Next day, the aged slides were treated with 0.010 M pepsin to remove cytoplasmic proteins, and then washed in 2×SSC (3 times) before baking at 65°C for 1 h. Probes were prepared by amplification of 10–100 ng of purified BAC DNA using GenomePlex Complete WGA Kit (Sigma) and subsequently ‘indirectly’ labelled with dinitrophenyl (DNP)-, digoxigenin- and biotin-dUTPs, respectively using the GenomePlex Reamplification kit (Sigma) with home-made 10×dNTP mixture, containing 2 mM each of dATP, dCTP and dGTP, together with 1.4 mM dTTP and 0.6 mM digoxigenin- or biotin-dUTP for digoxigenin- and biotin-dUTP labelling, or 1.6 mM dTTP and 0.4 mM DNP-dUTP for DNP labelling. For each three-colour FISH, approximately 100 ng each of DNP-, digoxigenin- and biotin-dUTP labelled DNA and approx 2 µg of snail genomic DNA (as repeat blocking agent) were precipitated using ethanol and then resuspended in 12 µl of hybridisation buffer containing 50% formamide, 10% dextran sulphate, 2×SSC, pH 7.0). Probe mixture was denatured prior to target DNA denaturation at 65°C for 10 min and allowed to re-anneal at 37°C for 20–30 min. Slides were denatured at 63°C in 70% formamide/30% 2×SSC for 90 s and then quenched in ice-cold 70% ethanol and subsequently dehydrated through a 70%, 90% and 100% ethanol series. Slides were air-dried before adding probes to the target areas, coverslips were applied and the edges of coverslips were sealed with Fixogum. The slides were left to hybridise in a humidity chamber at 37°C overnight. The following day, slides were detected using the appropriate antibodies after stringency washes in 50% formamide/50% 2×SSC at 42°C. The following reagents were used for detection. DNP was detected with (layer 1) rabbit anti-DNP- KLH IgG fraction (Invitrogen) and (layer 2) Alexa Fluor 488 conjugated donkey anti-rabbit IgG (Invitrogen). Digoxigenin was detected with (layer 1) Monoclonal ant-digoxin produced in mouse (Sigma) and (layer 2) Texas Red –×conjugated goat anti-mouse IgG (Invitrogen,). Biotin was detected directly with streptavidin-Cy3. After adding each layer of reagents, the slides were incubated for 15 min at 37°C incubator and then washed in 4×SSC containing 0.05% Tween-20 at 42°C, 3 times for 3 minutes each. After detection, slides were mounted with Slowfade Gold® antifade solution with 4′,6-Diamidino-2-Phenylindole (DAPI, Invitrogen).

To prepare extended chromatin fibres, tissues of snail ovotestis and liver were collected in PBS and a cell suspension made by dispersion with pipetting. The cell suspension was centrifuged for 5 min at 1200 rpm. The pellet was washed twice in PBS and a cell count was carried out. A suspension was made at a final concentration of approximately 2–3×10^6^ cells per ml. Chromatin fibres were made from live cells following previously published method [37] with modifications. Fibre slides were aged overnight in a sealed box with a desiccant and hybridisation was set up the following day. FISH onto fibre slides was carried out as described above for meiotic (pachytene) chromosomes.

FISH images were captured and processed using the SmartCapture® (Digital Scientific, UK) digital imaging system that consists of a Zeiss microscope (Axioplan 2 Imaging or AxioImager. D1) equipped with narrow bandpass filters for Cy3, Cy3.5, FITC (fluorescein isothiocyanate) and DAPI fluorescence, a cooled CCD camera (Hamamatsu) and an iMAC computer (Apple).

## Supporting Information

Table S1 **Summary statistics for RAD Sequencing library.** Raw Illumina read pair counts, candidate alleles and loci generated by RADtools, and mean coverage per allele for each of 10 dextral and 10 sinistral snails in the sequenced RAD-Seq library.
(XLSX)

Table S2 **Polymorphism in 20 putatively linked RAD-Seq loci.**
(XLSX)
